# The role of CD2AP in the Pathogenesis of Alzheimer's Disease

**DOI:** 10.14336/AD.2018.1025

**Published:** 2019-08-01

**Authors:** Qing-Qing Tao, Yu-Chao Chen, Zhi-Ying Wu

**Affiliations:** Department of Neurology and Research Center of Neurology in Second Affiliated Hospital, and Key Laboratory of Medical Neurobiology of Zhejiang Province, Zhejiang University School of Medicine, Hangzhou, China.

**Keywords:** Alzheimer’s disease, *CD2AP*, pathogenesis, genetics

## Abstract

Alzheimer’s disease (AD) is the most common neurodegenerative disease characterized by irreversible decline in cognition with unclear pathogenesis. Recently, accumulating evidence has revealed that CD2 associated protein (CD2AP), a scaffolding molecule regulates signal transduction and cytoskeletal molecules, is implicated in AD pathogenesis. Several single nucleotide polymorphisms (SNPs) in *CD2AP* gene are associated with higher risk for AD and mRNA levels of CD2AP are decreased in peripheral lymphocytes of sporadic AD patients. Furthermore, CD2AP loss of function is linked to enhanced Aβ production, Tau-induced neurotoxicity, abnormal neurite structure modulation and reduced blood-brain barrier integrity. This review is to summarize the recent discoveries about the genetics and known functions of CD2AP. The recent evidence concerning the roles of CD2AP in the AD pathogenesis is summarized and CD2AP can be a promising therapeutic target for AD.

Alzheimer’s disease (AD), the most common form of dementia, becomes more prevalent as the population ages [1]. Both genetic and environmental factors contribute to its risk. Amyloid precursor protein (*APP*), presenilin 1 (*PSEN1*), and presenilin 2 (*PSEN2*) are the major causative genes of familial AD (FAD) [2, 3]. Apolipoprotein E (*APOE*) ε4 allele has been consistently recognized to increase susceptibility to sporadic AD (SAD) [4]. During the last decades, genome-wide association studies (GWAS) have been identified a series of single nucleotide polymorphisms (SNPs) that associated with late-onset AD (LOAD) [5]. However, the mechanisms that lead to synapses degeneration and neuron death remain elusive. Investigations have identified multiple perturbations of cellular function in AD neurons, such as excessive amyloid protein (Aβ) deposition and neurofibrillary tangles, impaired mitochondrial function, abnormal calcium metabolism and altered axonal transport [6-9]. Several SAD risk genes have been studied in both cell and animal models, providing more insights into the cellular mechanisms underlying SAD neuron degeneration [10-12]. Recently, several studies reported that SNPs in the CD2 associated protein (CD2AP) gene were associated with SAD [13-15]. Furthermore, emerging evidence has demonstrated that CD2AP loss of function may play an important role in the SAD pathogenesis [16].

Here, we review the recent discoveries about the genetics and known functions of CD2AP. The recent evidence concerning the roles of CD2AP in the AD pathogenesis is summarized and CD2AP can be a promising therapeutic target for AD.

**Figure 1.**
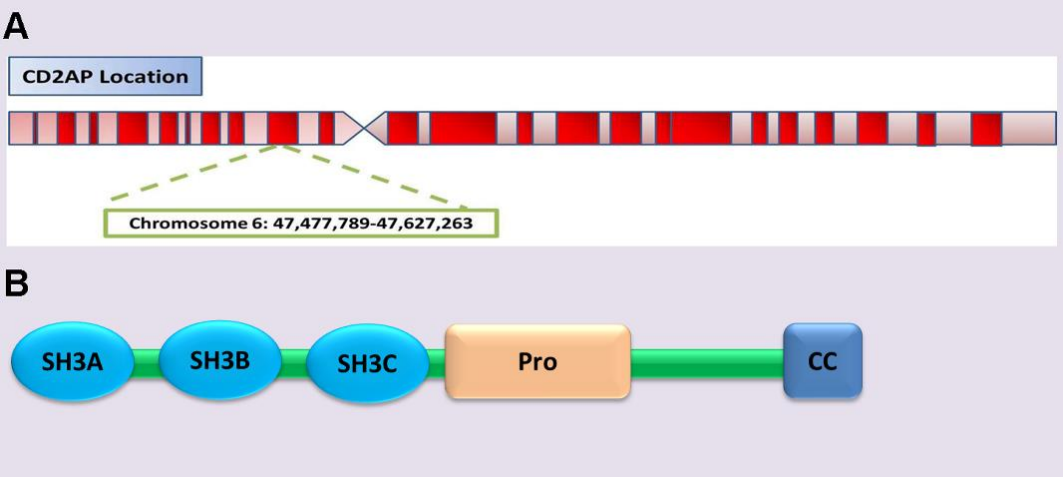
The chromosome location and schematic representation of the structural domains of *CD2AP* **A)** *CD2AP* is located on chromosome 6 (6p12.3). **B)** The protein has three consecutive SH3 domains at the amino terminus. The middle region is a proline-rich sequence. The carboxy terminus contains a duplex helical structural region where has a binding site for the actin cytoskeleton.

## Biochemical properties of CD2AP

CD2AP was first identified in 1998 and was named for its ability to bind to CD2 and promote CD2 aggregation to stabilize the interaction between T cells and antigen presenting cells [17]. *CD2AP* gene is located on chromosome 6 (6p12.3) and contains 18 exons. The encoded protein (CD2AP) is an adaptor protein consisting of 639 amino acid residues with a molecular weight of 80 KD. The protein has three consecutive SH3 domains at the amino terminus. Studies have shown that SH3 domains can interact with many signal transduction and cytoskeletal molecules. The middle region is a proline-rich sequence. The carboxy terminus contains a duplex helical structural region and has a binding site for the actin cytoskeleton. The chromosome location of *CD2AP* and schematic representation of the structural domains of CD2AP was shown in Figure 1. This protein has been well studied concerning its role in dynamic actin remodeling and membrane trafficking during endocytosis and cytokinesis [18].

CD2AP is ubiquitously expressed with higher levels in immune cells, epithelial cells as well as neurons. Previous studies have shown that CD2AP is necessary for signaling at the slit diaphragm of kidney [19-21]. Haploinsufficiency or homozygous mutation of the human CD2AP leads to renal disease [22]. Mice lacking CD2AP has phenotype of congenital nephrotic syndrome because of decreased podocytes foot process integrity and died of severe proteinuria at 6-7 weeks [23]. CD2AP has also been shown to associate with several scaffold and focal adhesion proteins such as F-actin and p130CAS [24, 25], supporting a critical role for CD2AP in specialized cell contacts. In addition, CD2AP was reported to directly bind to p53 protein in the cytoplasm [26]. Recently, a study has demonstrated that CD2AP in CD4 T cells modulates differentiation of follicular helper T cells during chronic lymphocytic choriomeningitis virus infection [27]. Another study has showed that CD2AP contributes to hepatitis C virus propagation and steatosis by disrupting insulin signaling [28].

## Genetics of *CD2AP* gene in AD

In 2011, Hollingworth et al. first reported in a large staged GWAS of AD that a specific SNP with *CD2AP* (rs9349407) located in intron 1, is significantly associated with AD risk in both stages (P1 = 8.0 ×10^-4^, OR1 = 1.11; P2 = 8.6 × 10^-9^, OR2 = 1.11, respectively) in Caucasian population [13]. Another study performed by Naj and colleagues using a 3-stage design GWAS also observed this result (rs9349407; P=8.6×10-9) [15]. After then, numerous replicated studies were performed to confirm this result. However, inconformity of the replication results occurred in a study of meta-analyses comprising 6 case-control series of 2634 LOAD and 4201 controls in Caucasian populations which found no relationship between *CD2AP* variant (rs9349407, OR 0.97, p=0.56) and LOAD [29]. Similarly, no association was found between rs9349407 or rs9296559 and LOAD risk in northern Chinese Han population and Korean population by Tan et al. and Chung et al., respectively [30, 31]. Recent year, another meta-analysis study selected 54, 936 subjects from East Asian, American, Canadian and European populations showed the significantly association between the SNP (rs9349407) of *CD2AP* and AD [32]. The studies performed to assess the association between rs9349407 and AD has been summarized by Chen et al. [32].

**Table 1:** Associations between *CD2AP* and sporadic AD.

Year	SNP ID	Source	Population	Cases/Controls	P value	OR	Association	Reference
2011	rs9349407	USA	African American	513/496	0.860	0.98	Negative	Logue et al. [38]
2011	rs9349407	Mayo2	American and European ancestry	2,521/4,055	0.560	0.97	Negative	Carrasquillo et al. [29]
2011	rs9349407	ADGC combined analysis (Stage 1+2)	European ancestry	11840/10931	1.00E-06	1.12	Positive	Naj et al. [15]
2011	rs9349407	GERAD+ consortia	European ancestry	6283/7165	8.00E-04	1.11	Positive	Hollingworth et al. [13]
2011	rs9296559	GERAD+ consortia	European ancestry	6283/7165	1.50E-03	1.1	Positive	Hollingworth et al. [13]
2013	rs9349407	North China	East Asian	612/612	0.850	1.024	Negative	Tan et al. [30]
2013	rs9349407	Japan	East Asian	891/844	0.380	0.94	Negative	Miyashita et al. [39]
2013	rs10948363	Four Consortia combined analysis (Stage 1+2)*	European ancestry	25580/48466	5.20E-11	1.1	Positive	Lambert et al. [34]
2013	rs9349407	America	American	725/651	0.029	NG	Positive	Shulman et al. [37]
2015	rs116754410	Toronto	Caucasian	330/333,70	5.33E-08	NG	Positive	Vardarajan et al. [35]
2015	rs9349407	South China	Han Chinese	229/318	0.048	1.368	Positive	Jiao et al. [36]
2015	rs10948363	South China	Han Chinese	229/318	0.395	1.138	Negative	Jiao et al. [36]
2017	rs9349407	Southeast China (Stage 1)	Han Chinese	422/1435	4.6E-10	2.11	Positive	Tao et al. [14]
2017	rs9296559	Southeast China (Stage 1+2)	Han Chinese	647/2,138	7.69E-09	1.773	Positive	Tao et al. [14]

* ADGC, CHARGE, EADI and GERAD; SNP: Single nucleotide polymorphism; NG: Not given; OR:odds ratio

Recently, our group performed a two-stage study showed that the C allele of rs9296559 increased the risk of SAD (P = 7.69×10-9, OR = 1.77) [14]. In addition, rs9349407 in *CD2AP* combined with rs11218343 in *SORL1*, rs17125944 in *FERMT2*, rs6859 in *PVRL2*, rs157580 and rs2075650 in *TOMM40* were used to calculate genetic risk score (GRS) in predicting SAD risk, and the results showed that the area under the receiver operating characteristic curve (AUC) for discriminating cases from controls was 0.58 for GRS, 0.60 for *APOE*, and 0.64 for GRS and *APOE* [33]. The associations between several other SNPs in *CD2AP* and SAD were also determined in a number of replicated studies [34-39]. The associations between SAD and SNPs in *CD2AP* were summarized in Table 1.

## Potential pathways underlying roles of CD2AP in AD

### Role of CD2AP in amyloidogenesis

CD2AP is expressed in neurons and capillaries in brain [40]. We previously reported the gene expression of* CD2AP* in peripheral blood lymphocytes was decreased in Chinese patients with SAD as compared with cognitively normal controls, implying CD2AP loss of function may implicate in SAD pathogenesis [14]. Aβ peptides are generated via sequential cleavage of APP by β-secretase and γ-secretase complexes during the course of its secretory pathway [41]. Several LOAD risk genes (*APOE*, *PICALM*, *BIN1*, *SORL1*, and *PLD3*) have been reported to implicate in Aβ42 production [42-46]. Liao et al. reported CD2AP could affect Aβ levels and Aβ42/Aβ40 ratio in vitro while its effects on Aβ metabolism were subtle in vivo [47]. As a part of adaptor protein complexes, CD2AP loss of function affects the sorting process in endosome-lysosome pathway. One study reported that glucose transporter 4 (Glut4) trafficking was impaired in podocytes lacking CD2AP [19]. In addition, CD2AP plays a role in maintaining early endosome morphology and the traffic between early and late endosomes [48]. A recent study demonstrated that CD2AP regulated Aβ generation by a neuron-specific polarization of Aβ in dendritic early endosomes. CD2AP could affect APP and BACE1 sorting in early endosomes by distinct mechanisms [12].

### Role of CD2AP in Tau-induced Toxicity

Abnormal phosphorylated Tau protein forms into neurofibrillary tangles in brain are one of the hallmarks of AD [9]. In a Drosophila model of AD, susceptibility genes that implicate in Tau-mediated mechanisms were screened. The results found cindr, the fly ortholog of the human CD2AP, was implicated as a modulator of Tau-mediated neurotoxicity [49]. Furthermore, cindr loss of function (cindr-/-) enhances Tau-induced neuronal loss in the adult fly brain. Significantly reduced survival times were observed in cindr-/- flies. In addition, cindr-/- flies also showed reduced synaptic strength and altered short-term plasticity [50].

### Role of CD2AP in other pathways underlying AD pathogenesis

CD2AP has been extensively studied in kidney podocytes. Lacking CD2AP leads to decreased mice podocytes foot process integrity [23]. Previous studies have reported that the podocyte major processes share lots of cell biological characteristics with neural dendrites [51]. Recently, a study revealed that CD2AP could modulate neurite length, neurite complexity, growth cone filopodia number in neurons, and these effects were in accordance with CD2AP expression levels. CD2AP regulate collateral sprouting and structural plasticity of intact adult axons by coordinate NGF signaling [52].

The blood-brain barrier (BBB) is a continuous endothelial membrane that separates the brain and extracellular fluid from the circulating blood in the central nervous system (CNS). The BBB breakdown and vascular degeneration plays a critical role in AD pathogenesis [53]. For example, *APOE* ε4-positive individuals have a reduced cerebral blood flow and increased BBB leakiness [54]. By contrast, individuals carrying *APOE* ε3, a protective factor of AD, show a decreased degree of BBB breakdown [55]. CD2AP is enriched in the brain microvascular endothelial cells, an essential component of BBB [17]. A recent study showed that CD2AP-deficient mice had reduced BBB integrity, suggesting cerebrovascular roles of CD2AP could take part into its role on AD risk. *CD2AP*-deficient mice had mild motor and anxiety deficits and showed more susceptible to pharmacologically induced seizures. No obviously other behavioral abnormalities were observed [56].

**Figure 2.**
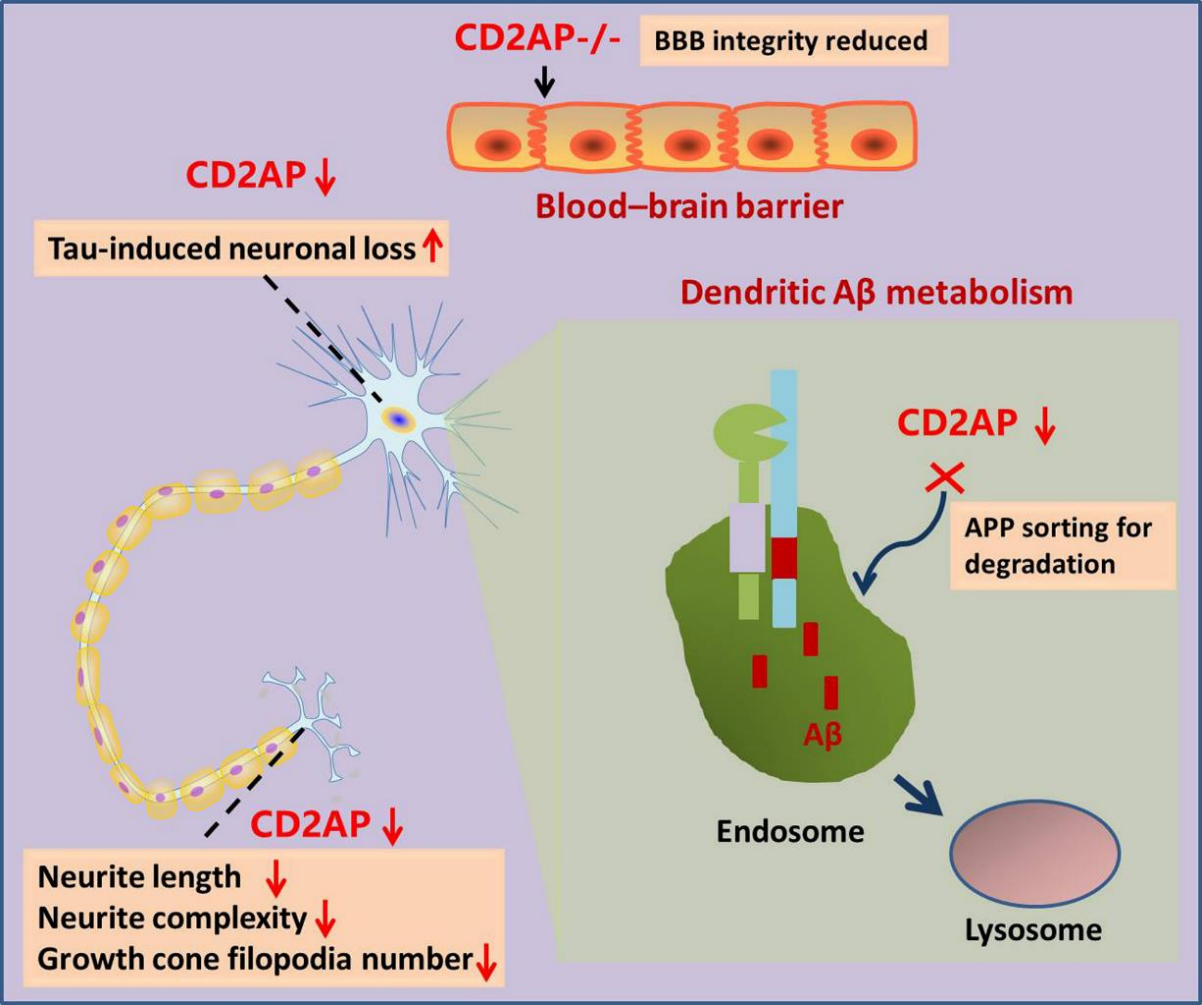
Possible mechanisms underlying CD2AP loss of function in the pathogenesis of sporadic AD CD2AP loss of function is linked to enhanced Aβ metabolism, Tau-induced neurotoxicity, abnormal neurite structure modulation and reduced blood-brain barrier integrity.

CD2AP and several other AD risk genes (*BIN1* and *PICALM*) were also predicated to participate in autophagy pathway [57, 58]. In CD2AP^-/-^ podocytes, one of the important proteins in autophagy signaling (p62) was upregulated, indicating the absence of CD2AP induced podocyte injury by affecting autophagy signaling. In addition, downregulation of the full-length caspase-1 was observed in podocytes lacking CD2AP [59]. However, the specific role of CD2AP in autophagy signaling was still elusive and need to be further addressed.

Neuro-inflammation is one of the pathological hallmarks of AD. Microglia is the main immune cell in the CNS. Accumulating evidences have revealed microglia dysfunction in both AD patients and mouse models [60-62]. CD2AP is expressed in both neuron and microglia in the brain and postulated to be involved in immune system regulation [63]. Identifying the role of CD2AP in microglia will help us better understand the pathogenesis of AD. The potential pathways underlying roles of CD2AP in the pathogenesis of AD were summarized in Figure 2.

## Conclusions

CD2AP is an adaptor protein that plays important roles in regulating signal transduction and cytoskeletal molecules. Although the association between *CD2AP* and higher risk of AD has been well addressed, the mechanisms of CD2AP implicated in AD pathogenesis still unclear. More and more evidence reveals that CD2AP loss of function results to enhanced Aβ metabolism, Tau-induced neurotoxicity, synapse dysfunction and abnormal neurite structure. Thus, targeting *CD2AP* may serve as a potential strategy for AD therapy. Elevating the expression of *CD2AP* in specific brain area could be a promising effective treatment. However, more accurate and detailed mechanisms by which *CD2AP* contributes to AD pathogenesis should be further explored.
